# Amyloidosis in Alzheimer's Disease: The Toxicity of Amyloid Beta (A**β**), Mechanisms of Its Accumulation and Implications of Medicinal Plants for Therapy

**DOI:** 10.1155/2013/413808

**Published:** 2013-05-15

**Authors:** Anchalee Prasansuklab, Tewin Tencomnao

**Affiliations:** ^1^Ph.D. Program in Clinical Biochemistry and Molecular Medicine, Department of Clinical Chemistry, Faculty of Allied Health Sciences, Chulalongkorn University, Bangkok 10330, Thailand; ^2^Center for Excellence in Omics-Nano Medical Technology Development Project, Department of Clinical Chemistry, Faculty of Allied Health Sciences, Chulalongkorn University, 154 Rama I Road, Pathumwan, Bangkok 10330, Thailand

## Abstract

Alzheimer's disease (AD) is a progressive neurodegenerative disorder that leads to memory deficits and death. While the number of individuals with AD is rising each year due to the longer life expectancy worldwide, current therapy can only somewhat relieve the symptoms of AD. There is no proven medication to cure or prevent the disease, possibly due to a lack of knowledge regarding the molecular mechanisms underlying disease pathogenesis. Most previous studies have accepted the “amyloid hypothesis,” in which the neuropathogenesis of AD is believed to be triggered by the accumulation of the toxic amyloid beta (A**β**) protein in the central nervous system (CNS). Lately, knowledge that may be critical to unraveling the hidden pathogenic pathway of AD has been revealed. This review concentrates on the toxicity of A**β** and the mechanism of accumulation of this toxic protein in the brain of individuals with AD and also summarizes recent advances in the study of these accumulation mechanisms together with the role of herbal medicines that could facilitate the development of more effective therapeutic and preventive strategies.

## 1. Introduction

Alzheimer's disease (AD) is a progressive neurodegenerative disorder named by the German physician Dr. Alois Alzheimer in 1906 [1]. This disease can occur in anyone at any age; however, it is most common among the elderly and is less prevalent in younger people. Although AD develops differently for each individual, it normally presents similar symptoms; in the early stages, the most common defect is remembering recent events or short-term memory impairment. As the disease progresses, AD patients gradually lose their ability to think and reason clearly, make judgments, solve problems, communicate, and take care of themselves. Symptoms also include confusion, irritability and aggression, mood swings, changes in personality and behavior, problems with attention and spatial orientation, trouble with language, and long-term memory loss, all of which can affect a person's daily life. AD can even lead to the death of the afflicted person in the final stages by causing malnutrition, brain death, and multiple organ failure due to the number of nerve cells that have died. At present, AD afflicts more than 26.6 million worldwide, and its prevalence is rising dramatically each year. By 2050, the number of AD patients is expected to quadruple to more than 106 million globally, and it is estimated that 1 in 85 persons will be living with the disease [2]. After several decades of study, AD is now considered as a complex disease that results from both genetic and environmental factors, such as age, gender, family history of AD, Down syndrome (DS), and the apolipoprotein E (apoE) gene. However, the actual causes of AD are still unknown. Additionally, the biochemistry of AD is not yet fully understood, even though its histopathological features in the brain are well characterized. So far, there have been a vast number of studies that have hypothesized disease mechanisms for AD, the majority of which support the amyloid hypothesis. It is believed that the neuropathogenesis of this disease may be triggered by the accumulation of toxic amyloid in the central nervous system (CNS). Therefore, a clearer understanding of how these toxic proteins accumulate in the brain of AD patients is significant for the development of more effective therapeutic and preventive strategies. Potential mechanisms related to overproduction or impaired clearance of these amyloids that may lead to its abnormal deposition in the brain as well as some possible molecular targets for AD treatment will be the focus of this review.

## 2. Amyloidosis

Amyloidosis is a large group of pathologic conditions in which a particular type of protein, called amyloid, is abnormally deposited in various tissues or organs. Generally, amyloid refers to misfolded peptides or proteins that demonstrate a stable, cross-beta super-secondary structure that renders it insoluble, fibrous-like, and resistant to proteolysis. Thus, amyloid may alter the normal function of tissues and cause serious changes in tissues and organs of the body [3, 4]. Amyloidosis can be inherited or acquired. In addition, the deposition of amyloid fibrils may occur in specific areas of a single tissue (localized amyloidosis) or throughout the body (systemic amyloidosis). Each type of amyloidosis is classified according to clinical signs and the main peptide or protein that constitutes the amyloid fibrils. Amyloidosis depots contain not only the major fibrillar component but also minor nonfibrillar components such as glycosaminoglycans (GAGs), apolipoprotein E (apoE), and serum amyloid P (SAP) components [3]. Despite the differences between amyloid proteins, all forms in different diseases share some common features; amyloid deposits exhibit an apple-green birefringence under a polarized light microscope after staining with the dye Congo red and appear as rigid, nonbranching fibrils 7.5 to 10 nm in diameter under extremely high magnification using an electron microscope [5]. To date, at least 28 different proteins have been identified as amyloids in humans [3, 5–7]. Several well-known examples of human amyloid-related diseases and the official nomenclature and classification of their causative agents are shown in Table 1. Alzheimer's disease (AD) and the toxicity and mechanisms of amyloid protein aggregation will be emphasized in this review.

## 3. Toxicity of Amyloids in Alzheimer's Disease

**Figure 1:**
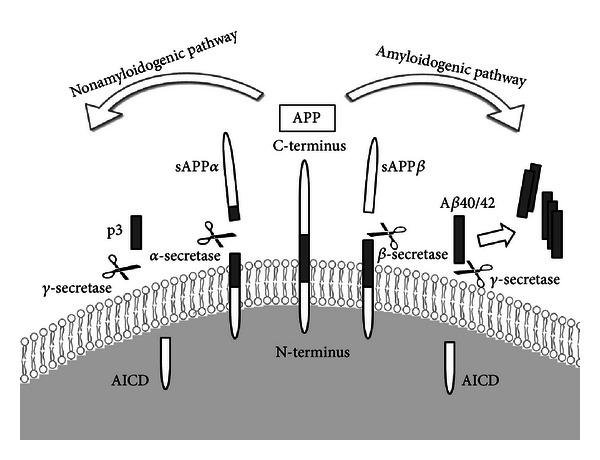
Proteolytic processing of amyloid precursor protein (APP). Amyloid precursor protein (APP) is a ubiquitously expressed integral membrane protein that can be processed in two distinct pathways. In the nonamyloidogenic pathway, APP is cleaved within the A*β* domain by the *α*-secretase enzyme. However, in the amyloidogenic pathway, APP is first cleaved by *β*-secretase (BACE1), instead of *α*-secretase, at the N-terminus of the A*β* domain, and this is followed by *γ*-secretase cleavage at the C-terminus. This sequence of events generates the A*β* amylogenic peptides, which can aggregate into oligomers and form extracellular neurotoxic plaques in the brain. Both pathways release identical APP intracellular C-terminal domain (AICD). This figure was adapted from Thinakaran and Koo (2008) [11].

The amyloid beta (A*β*) peptide was initially identified and biochemically characterized in 1984 [8] as a peptide that aggregated and was deposited outside neurons in the brain tissue of Alzheimer's patients, leading to the formation of neuritic plaques (also called senile or amyloid plaques) in the AD brain. The presence of these neuritic plaques is the major pathological hallmark of AD. The A*β* peptide, a principal component of these plaques, is thought to play a central role in AD and is regarded as the causative agent in development of the disease. This hypothesis has emerged from the fact that nearly all individuals with Down syndrome (DS), or trisomy 21, carry an extra copy of the amyloid precursor gene on chromosome 21. Therefore, their A*β* levels are high, and DS patients exhibit the clinical symptoms of AD around the age of 40 years [9, 10]. A*β* is a 4.2 kDa short peptide of 40–42 amino acids, generated from the intracellular cleavage of the amyloid precursor protein (APP) by the sequential action of two proteolytic enzymes, beta- (*β*-) secretase and gamma- (*γ*-) secretase. A schematic of the normal proteolytic processing of APP is shown in Figure 1.

APP is a single-pass transmembrane protein that is highly expressed in the brain and is concentrated at neuronal synapses [11, 12]. APP has been implicated in neuroprotection and as a regulator of neuronal cell growth, cell-cell, or cell-matrix interactions and synaptic plasticity [13]. However, its cleavage products from the amyloidogenic pathway can contribute to neurotoxicity. Indeed, soluble monomeric A*β* fragments are normally produced in the human body, but they can aggregate into various sized oligomers and insoluble fibrils, which subsequently form neuritic plaques. A*β* monomers are generated in most of the body's cells, including vascular endothelial cells [14], thyroid epithelial cells [15], and neuronal and nonneuronal cultured cells [16, 17]. However, neuronal cells seem to generate greater amounts of A*β* than other cell types [16], indicating that the A*β* peptide might play an important role in the normal physiology of the CNS. There is a notion that A*β* might serve as an essential synaptic protein in synaptic structural-functional plasticity underlying learning and memory, an idea supported by the increased long-term potentiation mediated by A*β*_40_ (LTP) [18]. Therefore, the neuropathological events occurring in individuals with AD likely result from the toxicity of amyloid oligomers and fibrils, which are the aggregated forms of A*β*, rather than from its monomeric form.

As A*β* accumulates, our bodies control the amyloid level through various mechanisms. In the normal brain, the concentration of the A*β* peptide is regulated by its production from APP and influx into the brain across the blood-brain barrier (BBB), mainly via the receptor for advanced glycation end products (RAGE), and by clearance from the brain via the low-density lipoprotein receptor-related protein-1 (LRP1) and enzymatic degradation within brain [19–21]. Thus, impairment of these regulatory mechanisms could lead to the accumulation and deposition of excessive amounts of A*β* peptide in the brain of individual with AD, the details of which are described later in this paper. 

The polymeric forms of A*β* trigger changes in biochemical molecules and functions in brain cells, resulting in several neuropathological abnormalities associated with the symptoms of AD. A*β* aggregate-mediated toxicity has been documented *in vitro* and *in vivo*. Initial reports in 1994 showed that elevated oxidative stress, one of the early pathological events of AD, was mediated by hydrogen peroxide (H_2_O_2_) produced through the reduction of metal ions by A*β* peptides [22–25]. This finding is consistent with the ability of A*β* to capture the transition metal ions Cu, Fe, and Zn, which are potent catalysts of oxidation [26]. These elements, particularly Zn, have also been implicated in promoting the oligomerization of A*β* peptides [27, 28]. Furthermore, extracellular and intracellular accumulation of metal ions is found in AD brains with high concentrations of A*β* plaques [29–32]. As a result of free radical production induced by A*β*, some biomolecules in the AD brain undergo conformational and structural changes due to lipid peroxidation and oxidative modification of proteins [33], leading to the dysfunction of these molecules and thereby influencing a wide array of cellular functions. Proteomic studies have identified several oxidatively modified proteins in AD [34, 35]. For example, oxidized ubiquitin carboxy-terminal hydrolase L-1 (UCH L-1) leads to proteasomal dysfunction and the consequent accumulation of damaged, misfolded, and aggregated proteins. Oxidatively modified creatine kinase BB (CK) and glutamine synthetase (GS) severely affect ATP production and the influx of calcium ions into neurons, resulting in the loss of function of ion pumps, the dysregulation of intracellular calcium homeostasis, alterations in LTP, and mitochondrial dysfunction with the release of proapoptotic factors. All of these changes could ultimately lead to neuronal death [36]. Increased oxidative stress, in turn, also promotes APP processing through the upregulation of BACE1 gene expression, which leads to an increase in AB generation [37–39]. 

Although the A*β* peptide plays an essential role at the synapse, A*β* aggregate-mediated toxicity impairs synaptic function, which leads to the progressive memory loss and cognitive failure associated with AD. Synaptic dysfunction is triggered by changes in synaptic structure and neurochemicals induced by oligomerized A*β* rather than amyloid plaques. Loss of synaptic terminals and LTP deficits have been demonstrated in studies of transgenic mice overexpressing mutant APP [40, 41]. Studies in the normal rodent hippocampus also showed that soluble A*β* oligomers isolated from the cerebral cortex of AD patients or from tissue culture reduced dendritic spine density and markedly inhibited LTP, resulting in the disruption of synaptic plasticity and memory [42, 43]. However, LTP was not impaired after treatment with insoluble amyloid plaque cores, suggesting that the oligomeric form rather than the deposit form is synaptotoxic. A*β* oligomer-induced synaptic dysfunction was found to be associated with a reduction in surface expression of both NMDA-type and AMPA-type glutamate receptors [44, 45] as well as postsynaptic density-95 (PSD-95) protein levels [46, 47] in cortical neurons; all three are key proteins in the postsynaptic density (PSD) involved in the regulation of synaptic function. Both glutamate receptors and nicotinic acetylcholine receptors (nAChRs) are also considerably reduced in the AD brain, possibly through A*β*-induced receptor internalization. The binding of exogenous A*β* to nAChRs facilitates internalization and intraneuronal accumulation of these toxic peptides, which could impact neuronal cells [48, 49]. Furthermore, a recent study proposed that activation of casein kinase II (CKII) by A*β* might underlie the disruption of synaptic transmission [50].

In addition to oxidative damage and synaptic failure, A*β* aggregates can induce mitochondrial dysfunction, which is another pathological hallmark of AD. The alteration of synaptic mitochondria, as a result of the buildup of A*β*, may underlie the synaptic pathology in AD [51]. Reported mitochondrial malfunctions as a consequence of membrane-localized A*β* include the inhibition of protein transport into mitochondria, the disruption of the electron transport chain leading to impaired glucose utilization in neurons, and mitochondrial damage due to an increase in reactive oxygen species (ROS) production [52]. A*β* also contributes to mitochondrial toxicity by the induction of microtubule-associated protein tau phosphorylation through specific kinase activation [53–55], which results in dissociation of tau from microtubules, the destabilization and disintegration of microtubules in axons, leading to the collapse of the neuronal transport system, and the formation of neurofibrillary tangles (NFTs) composed of aggregated hyperphosphorylated tau inside neuronal cell bodies. Moreover, proteasome-mediated degradation of misfolded protein, including tau aggregates, is inhibited by the actions of A*β* oligomers, contributing to their enhanced accumulation [56].

## 4. Risk Factors and Mechanisms Underlying Amyloid Beta Amyloidosis

**Figure 2:**
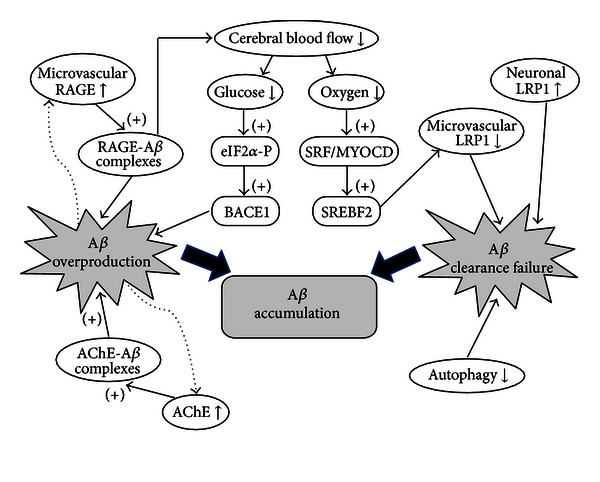
Schematic diagram of A*β* accumulation mechanism. This schematic diagram summarizes the factors influencing A*β* accumulation and the molecular mechanisms involved in the pathways for overproduction and impaired clearance of toxic A*β* peptides. Several potential targets for AD treatment, such as AChE, LRP1, and RAGE, and certain components involved in the control of cerebral blood flow and the autophagic pathway are suggested by their direct involvement in A*β* accumulation mechanism.

Because the amyloid hypothesis postulated that accumulation of A*β* is the fundamental cause of AD, factors that lead to excessive levels of A*β* have been investigated in a number of studies, especially those factors associated with the pathway for overproduction and impaired clearance of amyloids. This review summarizes the latest information on factors influencing A*β* levels, as well as the molecular mechanisms involved in the above-mentioned pathways, including the schematic diagram of A*β* accumulation mechanisms influenced by these factors in Figure 2.

### 4.1. Cholinergic System

Cholinergic signaling was the primary factor described in the oldest AD hypothesis, which states that the progression of AD is initiated by a deficiency in the production of the vital neurotransmitter acetylcholine. Amyloid peptide was also found to be involved in this hypothesis [68] and may play a central role in producing the cholinergic deficit, as suggested by Ehrenstein et al. (1997). Amyloid peptide reduces acetylcholine (ACh) synthesis through A*β*-induced leakage of choline across cell membranes [69]. Moreover, A*β* was shown to affect nAChR levels [48]. In turn, the loss of ACh was reported to be associated with the production of A*β*. However, how the decline in ACh is linked to the increased level of A*β* has remained unclear. A known link involves acetylcholinesterase (AChE), the activity of which is increased around amyloid plaques, although it is lower in other regions of the AD brain [70, 71]. These findings were supported by later studies of the effect of A*β* on AChE expression [72, 73]. It was demonstrated that AChE could promote A*β* fibril and plaque formation both *in vitro* and *in vivo* [74, 75], which is caused by the interaction of A*β* peptide with AChE at a specific motif located close to the peripheral anionic binding site (PAS) of the enzyme [76]. Furthermore, AChE-A*β* complexes are more neurotoxic than A*β* alone, which is related to changes in components of the wingless-type MMTV integration site family (Wnt) signal transduction pathway. The level of beta-catenin, a key component of Wnt signaling, was found to be decreased through the action of AChE-A*β* complexes, whereas enhancing this pathway by lithium could block AChE-A*β*-dependent neurotoxicity [77–79]. In fact, one type of currently available drug, such as donepezil hydrochloride, a reversible competitive inhibitor of AChE, targets AChE. Unfortunately, it is not able to cure AD but reduces the symptoms for a limited period of time.

### 4.2. Receptor for Advanced Glycation End Products (RAGEs)

RAGE is a member of the immunoglobulin superfamily of cell surface molecules, which is able to recognize a variety of ligands such as advanced glycation end products (AGEs), HMGB1 (amphoterin), S100 protein, macrophage-1 antigen (Mac-1), and amyloid protein. The interaction between A*β* and RAGE in brain vessels mediates the transportation of circulating A*β* peptides across the BBB into the brain [20, 21, 80]. Expression of RAGE is determined by the levels of its ligands; thus, high production of A*β* results in the upregulation of RAGE, which in turn leads to the greater accumulation of toxic proteins in the brain. Thus, targeting RAGE might be beneficial for AD treatment. A study of human brains using western blotting and immunostaining analyses revealed elevated RAGE levels in the AD hippocampus [81]. In addition, a RAGE-A*β* interaction has been implicated in AD pathogenesis, and previous studies have shown that increased RAGE expression is associated with neurotoxicity. The interaction of A*β* with vascular RAGE enhanced the expression of proinflammatory cytokines and potent vasoconstrictor endothelin-1 (ET-1), which may result in decreased cerebral blood flow [20]. Insufficient blood flow was also reported to be associated with A*β* accumulation, and these details are discussed below.

### 4.3. Low-Density Lipoprotein Receptor-Related Protein-1 (LRP1)

LRP1 (also known as apolipoprotein E (apoE) receptor) is a member of the low-density lipoprotein (LDL) receptor family that is highly expressed in the CNS and plays a critical role in brain lipoprotein metabolism, including clearance of amyloid peptides [82]. The interaction between A*β* and LRP1 may mediate transport of A*β* out of the brain. Approximately, 70 to 90% of circulating plasma A*β* is normally controlled by the soluble form of this receptor, soluble LRP1 (sLRP1) [83]. However, whereas LRP1 along brain capillaries showed amyloid clearance capacity [84], LRP1 expressed in neurons is not likely responsible for the removal of A*β* but may instead promote neuronal uptake of A*β* via its endocytic function [85]. A study in transgenic mice overexpressing functional LRP1 receptors showed an increase in soluble brain A*β* accumulation [86]. This is consistent with the high LRP1 concentrations in neurons reported in the study of human AD hippocampi, while minimal LRP1 levels were found in microvessels of AD cases [81]. Hence, expression of capillary endothelial LRP1 and neuronal LRP1 is implicated in A*β* clearance and leads to toxic amyloid accumulation in AD brain; thus, LRP1 could be another potential target for AD treatment.

### 4.4. Autophagy

Autophagy is a lysosomal degradation pathway for the turnover of cytoplasmic components and aggregated proteins, including dysfunctional organelles. Autophagy is essential for maintaining cellular homeostasis. It has been suggested that alteration in autophagic processing is linked to AD pathogenesis due to its relevance in the removal of toxic A*β* aggregates as well as APP [87]. In addition, autophagy-induced A*β* production activity was implicated in a new pathway for APP processing [88]. Enhancing the autophagic degradation pathway was shown to protect neurons from A*β*-induced neurotoxicity, which was in turn increased by transcriptional silencing of the autophagic gene (Atg) [89]. Therefore, impaired autophagy could lead to A*β* accumulation. For that reason, modulation of this pathway might be a potential AD therapy. The Ser/Thr kinase mammalian target of rapamycin (mTOR), which plays a central role in autophagic regulation, or other components of the mTOR signaling pathway, could be efficient therapeutic targets for AD.

### 4.5. Cerebral Blood Flow

Recently, poor blood flow was suggested to be a main cause of AD. Energy and oxygen starvation in the brain resulting from insufficient blood flow potentially initiate the signaling pathways influencing A*β* biosynthesis and the brain's ability to remove this toxic protein. O'Connor et al. (2008) showed that BACE1 is regulated in response to stress from energy deprivation at the translational level [90]. An insufficient supply of glucose induces phosphorylation of the translation initiation factor eIF2alpha (eIF2*α*), which consequently increases BACE1 levels, resulting in overproduction of A*β*. In parallel, a study by Bell et al. (2009) reported that hypoxia also causes the impairment of brain clearance of A*β* through stimulation of serum response factor (SRF) and myocardin (MYOCD) expressions [91], which were found to be much more active in the blood vessels of brains of people with AD than in people who do not have the disease [92]. Overexpression of these two proteins in cerebrovascular smooth muscle cells (CVSMCs) negatively regulates the expression of LRP1, which is a key A*β* clearance receptor in the BBB, by stimulating the transactivation properties of the sterol regulatory element binding transcription factor 2 (SREBF2), ultimately leading to toxic A*β* accumulation. Therefore, the components of signaling pathways underlying reduced blood flow might be potential targets for AD treatment. Improving blood flow by exercise, healthy eating, or using dietary supplements may also be effective for preventing AD. Substantial evidence demonstrates an association between physical activity and improvement of cognitive decline in AD [93–95].

## 5. Therapeutic Perspectives of Herbal Medicine in AD

While the number of individuals with AD is rising each year due to a longer life expectancy worldwide, there is currently no drug treatment that provides a cure for AD. The currently available medications only relieve the symptoms of AD. Drugs commonly used to treat AD include AChE inhibitors, such as donepezil hydrochloride (Aricept), rivastigmine (Exelon), and galantamine (Reminyl). All three AChE inhibitors are reversible inhibitors of AChE and interact with the active site of AChE to prevent the breakdown of the vital neurotransmitter ACh, thereby allowing a higher level of ACh in the brain. Another drug, memantine (Ebixa), is an antagonist of the NMDA-type glutamate receptor. The action of memantine is quite different from that of the three AChE inhibitors. Memantine prevents neuronal cell death due to glutamate receptor overstimulation. Recently, selective monoamine oxidase B (MAO-B) inhibitors (MAOIs) were developed as agents for AD therapy [96]. MAOIs act by inhibiting the activity of the metabolizing enzyme MAO-B to prevent the breakdown of monoamine neurotransmitters, thus increasing their availability. However, these drugs are not effective for everyone with AD and can only temporarily slow down the progression of symptoms. Some users also experience adverse drug reactions or side effects. Therefore, developing alternative treatments for AD is needed. 

One type of alternative treatment that could be effective in curing AD or preventing the disease is herbal medicine. Using natural compounds from plants for medication is becoming more popular because of their wide availability, low cost, and potential for fewer adverse reactions than synthetic drugs. Nevertheless, the safety and efficacy of each plant or natural product must be confirmed before human usage. Many herbs have been reported to exhibit a neuroprotective effect in AD. Herbal medications targeting the mechanisms underlying A*β* accumulation, which is now believed to be a central causative pathway in AD pathogenesis, might be the most effective approach to preventing the disease. For example, cerebral blood flow-modulating plants may be beneficial. The ethanolic extract of the *Morinda citrifolia* fruit, including its chloroform and ethyl acetate fractions, was recently reported to significantly improve cerebral blood flow in a mouse model, suggesting that *M. citrifolia* may prevent A*β* accumulation. Interestingly, increased oxidative stress and AChE activities, common problems in AD, were also attenuated by the ethanolic extract of *M. citrifolia*, which supports its potential to prevent AD [97, 98]. Affecting the regulation of expression of genes involved in amyloidogenesis may be another mechanism of neuronal protection by plants. The components of *Caulis piperis futokadsurae* were reported to selectively inhibit the expression of the APP gene [99]. The extracts from several traditional Chinese herbs such as *Astragalus membranaceus *[100], *Paeonia suffruticosa *[101], *Magnolia officinalis *[102], and* Rhizoma anemarrhenae *[103] were reported to effectively prevent memory impairment via downregulation of the expression or activity of BACE1, thereby reducing APP levels in animal models. Inhibition of BACE1 activity was also reported in an *in vitro* study using extracted components of *Panax notoginseng* [104] and *Polygala tenuifolia* [105]. Autophagy-regulating plants might also help to prevent AD by altering A*β* clearance through the autophagic process. There are reports, both *in vivo* and *in vitro*, that alkaloids isolated from *Stephaniae tetrandrae* [106] could induce the expression of microtubule-associated protein-1 light chain 3 (LC3) and autophagy-related gene 7 (Atg7), which promote autophagy and the removal of A*β*. *Glycyrrhiza glabra* root extract [107], including *β*-Elemene, an active component derived from herbs used in traditional Chinese medicine [108], induces autophagy by increasing the levels of the LC3 protein. Additionally, curcumin, a major active component of *Curcuma longa* (turmeric), has been proposed to be a promising candidate for treatment of AD as shown by the enhanced clearance toxic A*β* in a number of studies [109–111], although the mechanisms of action of curcumin in AD are still unclear. Our review suggests that the autophagic mechanism, which has recently been identified as a target of curcumin [112–114], may be responsible for its potent antiamyloidogenic effects. Several previous studies have supported the effectiveness of herb extracts to treat AD by influencing A*β* accumulation. Interestingly, recent studies from our laboratory also provided evidence of the medicinal uses of plants for AD protection. The ethanolic extracts of *Rhinacanthus nasutus* leaf and root showed a beneficial effect protecting against the neuronal cell death induced by A*β* treatment or hypoxia in a cell culture study [115, 116]. The molecular mechanisms underlying this therapeutic effect need to be investigated further.

In conclusion, AD is a progressive neurodegenerative disorder that leads to memory impairment and death. However, there is currently no proven medication to cure or stop the progression of the disease. This review focused on the “amyloid hypothesis,” which states that the neuropathogenesis of AD is triggered by the accumulation of toxic A*β* in the CNS. We highlighted the importance of medicinal plants as alternative therapeutic or preventive agents for AD in the near future.

## Figures and Tables

**Table 1:** Some examples of amyloid proteins, their functional precursors, and related diseases.

Amyloid protein (abbreviation)	Precursor protein	Disease	Distribution/type	Reference
A*β*	A*β* precursor protein	Alzheimer's disease	Localized/hereditary or Acquired	[57]
A*β*	A*β* precursor protein	Cerebral amyloid angiopathy	Localized/hereditary	[58]
AIAPP	Pro-IAPP	Diabetes mellitus type II	Localized/???	[59]
AL	Immunoglobulin light chain	Primary systemic amyloidosis	Systemic/acquired	[60]
AA	Serum amyloid A	Rheumatoid arthritis	Systemic/acquired	[61]
ATTR	Wild-type transthyretin	Senile systemic amyloidosis	Systemic/acquired	[62]
ATTR	Transthyretin variant	Familial amyloid polyneuropathy	Systemic/hereditary	[63]
AFib	Fibrinogen *α*-chain variant	Familial amyloidosis	Systemic/hereditary	[64]
A*β*2M	Beta 2 microglobulin	Hemodialysis-associated amyloidosis	Systemic/acquired	[65]
APrP^SC^	Prion protein	Creutzfeldt-Jakob disease	Localized/hereditary	[66]
AANF	Atrial natriuretic factor	Isolated atrial amyloidosis	Localized/acquired	[67]
